# Current Role of Minimally Invasive Radical Cholecystectomy for Gallbladder Cancer

**DOI:** 10.1155/2016/7684915

**Published:** 2016-11-03

**Authors:** Giuseppe Zimmitti, Alberto Manzoni, Francesca Guerini, Marco Ramera, Paola Bertocchi, Francesca Aroldi, Alberto Zaniboni, Edoardo Rosso

**Affiliations:** ^1^Department of General Surgery, Istituto Ospedaliero Fondazione Poliambulanza, Via Bissolati n 57, Brescia, Italy; ^2^Department of Oncology, Istituto Ospedaliero Fondazione Poliambulanza, Via Bissolati n 57, Brescia, Italy

## Abstract

*Background*. For Tis and T1a gallbladder cancer (GbC), laparoscopic cholecystectomy can provide similar survival outcomes compared to open cholecystectomy. However, for patients affected by resectable T1b or more advanced GbC, open approach radical cholecystectomy (RC), consisting in gallbladder liver bed resection or segment 4b-5 bisegmentectomy, with locoregional lymphadenectomy, is considered the gold standard while minimally invasive RC (MiRC) is skeptically considered.* Aim*. To analyze current literature on perioperative and oncologic outcomes of MiRC for patients affected by GbC.* Methods*. A Medline review of published articles until June 2016 concerning MiRC for GbC was performed.* Results*. Data relevant for this review were presented in 13 articles, including 152 patients undergoing an attempt of MiRC for GbC. No randomized clinical trial was found. The approach was laparoscopic in 147 patients and robotic in five. Conversion was required in 15 (10%) patients. Postoperative complications rate was 10% with no mortality. Long-term survival outcomes were reported by 11 studies, two of them showing similar oncologic results when comparing MiRC with matched open RC.* Conclusions*. Although randomized clinical trials are still lacking and only descriptive studies reporting on limited number of patients are available, current literature seems suggesting that when performed at highly specialized centers, MiRC for GbC is safe and feasible and has oncologic outcomes comparable to open RC.

## 1. Introduction

The role of laparoscopic surgery in the management of digestive tract tumor is increasingly accepted worldwide [1, 2]. However, although laparoscopic cholecystectomy (LC) began the era of laparoscopic surgery and is one of the most frequently performed mini-invasive procedures, the use of laparoscopic surgery is skeptically considered in the management of gallbladder cancer (GbC) [3]. GbC represents the most aggressive malignancy of the biliary tract and is characterized by an extremely poor prognosis. While increasing evidence shows that, for Tis and T1a GbC with clear margins and unbroken gallbladder, simple cholecystectomy, either laparoscopic or open, can be curative [4–7], for patients affected by resectable T1b or more advanced GbC, radical cholecystectomy (RC), consisting in liver resection (liver bed resection or segment 4b-5 bisegmentectomy) with locoregional lymphadenectomy, is the only available treatment to positively affect the prognosis [8–12].

Minimally invasive RC (MiRC) is skeptically considered by the majority of HPB surgeons, mainly due to the fear of tumor dissemination during laparoscopy, to difficulty in achieving adequate lymphadenectomy, and to complexity of laparoscopic liver resection.

The aim of this study is to review the available literature on feasibility and postoperative and oncologic outcomes of MiRC for patients affected by T1b or more advanced GbC.

## 2. Materials and Methods

Clinical case studies reporting on patients affected by GbC who underwent MiRC, meaning that laparoscopic or robot-assisted approach was used for both liver resection and locoregional lymph nodes excision, were included in the current review. Case reports, case series of MiRC, and case-control studies of MiRC versus open approach RC were reviewed. We included studies describing MiRC both elective, that is, performed when GbC was suspected before cholecystectomy, and revisional, that is, performed as a completion treatment after a GbC was diagnosed following a simple cholecystectomy. Articles reporting on patients undergoing simple LC for GbC, as well as those undergoing minimally invasive locoregional lymphadenectomy without liver resection, were excluded from this analysis. We systematically searched Medline (through PubMed) [13, 14] for all years to June 2016 (last PubMed search was performed on June 10, 2016).

Initially, searches employing MeSH terms were performed for keywords and text (title or abstract). As shown in Table 1, search terms were organized in three main groups (search number: 3, 11, and 12), which were further combined with each other finally resulting in the identification of 242 manuscripts. In addition, a “manual” research using the “related articles” function was used in order to “explode” research, and results were supplemented by further searches of reference lists of other articles, resulting in the identification of additional 24 manuscripts. Titles, abstracts, and full texts of resulting 266 manuscripts were independently reviewed by two authors (GZ and AM) to assess whether the studies met the eligibility criteria. Contrasting results between GZ and AM were discussed case by case, until an agreement was found. Included articles could be classified in case reports and series of MiRC for GbC and case-control studies comparing results of MiRC versus open radical cholecystectomy. An intention-to-treat analysis was performed; consequently, cases converted to open procedures were included in the analysis.

## 3. Results

**Figure 1:**
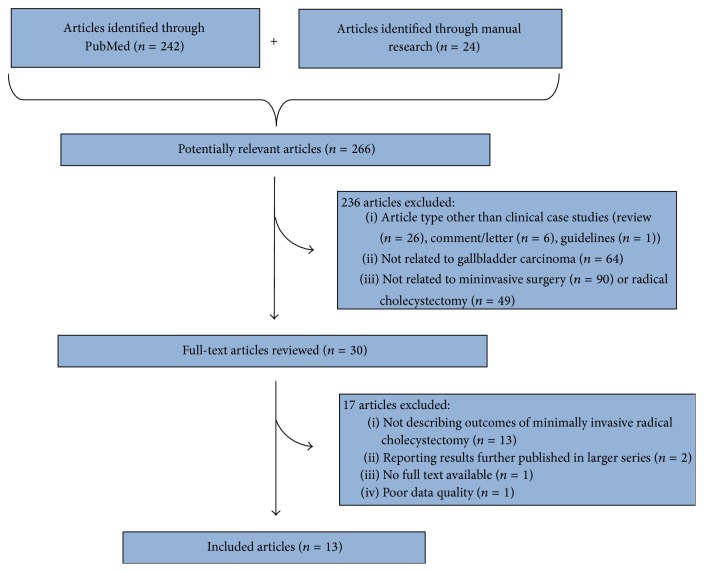
Strategy for article search and selection.

According to the aforementioned criteria, of 266 manuscripts identified by Medline (through PubMed) and by manual research, 236 were initially excluded by title or abstract analysis, leading to 30 articles. Such articles, if available, were further reviewed by full text analysis, finally leading to 13 articles [15–26,13] whose content was considered relevant for the current review (Figure 1).  Table 2 shows characteristics of articles included in our study.

Of 168 patients included in the 13 aforementioned studies, 16 underwent a LC according to study protocol [22, 27]. Of the remaining 152 patients who underwent an attempt of MiRC, minimally invasive approach was laparoscopic in 147 patients and robotic in 5 patients. Overall, 15 (10%) patients were converted to an open procedure according to study protocol in one case [17], due to postcholecystectomy adhesions which made peritoneal laparoscopic exploration unfeasible in 11 cases [17], due to intraoperative portal bleeding in one case [21], and due to intraoperatively detected persisting bile leak from the liver bed in the remaining case [17]. MiRC was attempted as an elective procedure for a preoperative suspicion of GbC in 110 patients and as a completion procedure following diagnosis of GbC on a cholecystectomy specimen in the remaining 42 patients. MiRC included at least a resection of the liver bed in all but 5 patients who, according to the corresponding study protocol [23], underwent simple cholecystectomy because the GbC was located on the peritoneal side of the gallbladder. Liver resection consisted in liver bed resection in 98 patients (with a liver bed thickness ranging between 2 mm [22] and 3 to 5 cm [18, 19]), segment 4b-5 resection in 49 patients [15, 24, 26], and extended right hepatectomy in 2 patients [21]. Laparoscopic intraoperative ultrasonography was performed during MiRC in seven studies. Locoregional nodal excision was performed in all patients and represented the initial resective procedure in 7 studies. Port site excision was performed in 8 out of 42 (19%) patients who underwent completion MiRC (Table 3).

Mean intraoperative blood loss was 150 cl (range: 0–1500 cl); mean operation duration was 235 minutes (89–490 min). Overall, 15 patients (10%) out of 152 who underwent an attempt of MiRC experienced postoperative morbidity, while postoperative mortality was nil. Following MiRC, mean length of hospital stay was 5 (2 to 19) days. Final pathology data were available for 144 patients and revealed T0-1a, T1b, T2, and T3 GbC in 9, 36, 81, and 18 cases, respectively. Mean retrieved lymph node number ranged between three and 13; lymph node status was N0 in 115 patients, N1 in 21, and NX in the remaining 8. R0 resection was obtained in all patients undergoing MiRC. Long-term survival outcomes were reported in 11 studies: after a mean follow-up duration ranging between 11 and 84 months, 14 patients experienced disease recurrence, whose location was specified in 10 cases. No port site recurrence was observed during follow-up (Table 4).

## 4. Discussion

In the current review, we retrospectively analyzed perioperative and oncologic outcomes of 152 patients, from the 13 studies reporting on MiRC for GbC available in PubMed up to June 2016. Despite the absence of randomized clinical trial comparing results of MiRC with open RC and the limited number of patients included in this review, current evidence seems to support MiRC, both in an elective setting, when RC is performed in case of suspected GbC before cholecystectomy is performed, and in a completion setting, meaning that RC is performed after GbC has been incidentally diagnosed on a cholecystectomy specimen. Available studies report low rates of conversion to open procedure and of intraoperative complication for MiRC with limited intraoperative blood loss, paralleled by nil mortality, acceptable morbidity rates, and short length of stay following surgery, making MiRC feasible and safe. In addition, two comparative studies reported a comparable number of retrieved lymph nodes and a comparable survival rate between MiRC and open RC, supporting oncological validity of MiRC [26, 27].

The ideal surgical management of patients affected by GbC is related to the tumor stage, being simple cholecystectomy sufficient for patients affected by Tis or T1a tumor. In this context, increasing scientific evidence has shown that oncologic outcomes of LC are similar to open cholecystectomy for Tis and T1a GbC [7]. In contrast, for patients affected by T1b or higher stage tumor necessitating RC, a minimally invasive approach is questioned by majority of HPB surgeons.

Skepticism concerning MiRC is mainly related to historical studies which have previously associated tumor recurrence with laparoscopic approach among patients affected by incidental GbC, undergoing laparoscopic cholecystectomy [29–32]. In particular, reports concerning port site recurrence and peritoneal dissemination of cancer cells [33–40] brought about a cautionary note about the use of laparoscopy in patients in whom GbC was suspected, making GbC a formal contraindication for laparoscopy. Additional reports correlated the occurrence of port sites and peritoneal implantation to the association of CO_2_ pneumoperitoneum effect and imprecise handling of gallbladder during laparoscopy leading to accidental perforation of gall bladder [36, 40–42]. Port site and peritoneal recurrence may occur through direct and indirect implantation of tumor cells, during the laparoscopic procedure [43]. Direct implantation may depend on seeding of exfoliated malignant cells during tumor extraction without a protective bag or on contact with instruments contaminated with tumor cells. Indirect contamination may be related to pneumoperitoneum, based on an “aerosol” effect with dissemination of exfoliated tumor cells to the port sites during the turbulence of insufflation or to a “chimney” effect with tumor cells wound implantation during desufflation [44]. However, increasing evidence highlights the role of gentle manipulation of gallbladder and of the use of plastic bag for specimen extraction in reducing the rate of port site and peritoneal tumor implantation [23, 24, 40], while pneumoperitoneum* per se *seems to have a limited role in the development of such complications. Consistent with this hypothesis, we currently found that, among 10 patients whose disease recurrence following MiRC was specified in detail, no peritoneal or port site recurrence occurred, further supporting the hypothesis that laparoscopic approach is not directly responsible for increasing the risk of peritoneal and port site dissemination, provided that gallbladder wall is not damaged and GbC is not exposed during MiRC.

RC is a complex procedure, consisting in liver bed or more extended anatomic liver resection, associated with hepatic pedicle lymph node dissection, eventually extended to peripancreatic and para-aortic lymph nodes, and, eventually, in selected cases, to common bile duct resection and reconstruction. Intrinsic technical difficulty of performing such procedures by minimally invasive approach represents an additional factor slowering MiRC acceptance. Concerning liver resection, the technique of laparoscopic liver resection has already been established [45], and radical laparoscopic surgeries for liver diseases have been demonstrated to show outcomes equal to those of open surgeries [46, 47]. Although the thickness of liver parenchyma to resect during RC remains a matter of debate, current evidence supports safety and feasibility of a minimally invasive approach for liver bed resection, as well as for segmentectomy 4b-5.

GbC has a high tendency to lymphatic invasion; thus, an adequate lymphadenectomy, commonly identified as the retrieval of at least six lymph nodes, is required to obtain a proper tumor staging [10, 38, 48]. Hepatic portal pedicle is a complex structure, containing important and delicate elements whose damage during lymphadenectomy may result in uncontrollable bleeding or injury to bile duct. This has brought about question of safety and adequacy of laparoscopic lymphadenectomy for GbC, representing an obstacle to the advancement of MiRC for GbC. However, current evidence suggests that laparoscopic lymphadenectomy may yield outcomes similar to those following open approach RC, with a mean number of dissected lymph nodes ranging between three [18, 19] and 13 [23, 27] among 13 studies analyzed in the current review. In addition, two studies [26, 27] comparing results of MiRC and of open RC did not show statistical difference between two approaches concerning the mean number of lymph nodes retrieved.

Finally, concerning bile duct resection and reconstruction, this can be indicated during RC only if the cystic duct margin is positive and although in some of the analyzed studies such situation represented an indication to open RC [22, 27], it does not represent an absolute contraindication to MiRC. Two of the 13 studies reviewed in the current analysis report on overall 3 patients who underwent a MiRC associated with common bile duct resection and biliojejunal reconstruction, none of them developing postoperative complications related to the procedure.

## 5. Conclusions

In conclusion, available data on MiRC for GbC show encouraging results in terms of perioperative and oncologic outcomes. However, the limited number of descriptive studies reporting on patients undergoing MiRC, as well as the absence of randomized clinical trials comparing MiRC and open RC, does not allow for recommending MiRC outside highly specialized centers with adequate experience in hepatobiliary and laparoscopic surgery.

## Figures and Tables

**Table 1:** Search strategy for Medline.

Search number	Search term	Results
1	Mininvasive surgical procedures [MeSH]	411011
2	Laparoscopy [MeSH]	77653
3	1 OR 2	411011
4	Hepatectomy [MeSH]	24427
5	Liver resection	46716
6	Hepatic resection	15180
7	Segmentectomy	8995
8	Radical cholecystectomy	479
9	Extended cholecystectomy	419
10	Lymphadenectomy	47649
11	4 OR 5 OR 6 OR 7 OR 8 OR 9 OR 10	56877
12	(Gallbladder OR Gall bladder) cancer OR tumor OR carcinoma OR neoplasm	13250
13	3 AND 11 AND 12	242

**Table 2:** Type of articles included in this review.

Type of article	Number	References
Case report	1	[15]

Case series of MiRC	10	[16]
		[17]
		[18]
		[19]
		[20]
		[21]
		[22]
		[23]
		[24]
		[25]

Case-control studies of MiRC versus open RC	2	[26]
		[27]

**Table 3:** MiRC for GbC: surgical technique details.

Author, year of publication	Study period	Patients number	Attempted MiRC	Conversion to open RC, rate	Completion/ elective	Liver resection	LIOUS	Liver transection technique	Type of liver resection	LB thickness	Nodal excision	Port site excision	Nodal excision first	Pringle	CBD resection (patients number)
Cho et al., 2008 [16]	NS	3	3	0	0/3	Yes	Yes	Harmonic scalpel, clips	LB	2 cm	Yes	—	Yes	No	No
de Aretxabala et al., 2010^*∗*^ [17]	2005–2009	18	18	13/18	18/0	Yes	No	Harmonic scalpel, clips, vascular stapler	LB	NS	Yes	No	Yes	Only if needed	No
Gumbs and Hoffman, 2010 [18]	NS	3	3	0	1/2	Yes	Yes	Harmonic scalpel, bipolar, clips	LB	3–5 cm	Yes	1/1	Yes	No	No
Gumbs and Hoffman, 2010 [19]	NS	6	6	0	0/6	Yes	Yes	Harmonic scalpel, bipolar, clips	LB	3–5 cm	Yes	—	Yes	No	Yes (1)
Belli et al., 2011 [25]	2006–2008	4	4	0	4/0	Yes	NA	NA	Wedge	2 cm	Yes	Yes	No	Yes	0
Shen et al., 2012^*£*^ [20]	2010-2011	5	5	0	2/3	Yes	No	Harmonic scalpel, electric hook	LB	2 cm	Yes	No	No	No	No
Gumbs et al., 2013^#^ [21]	2005–2011	15	15	1/15	5/10	Yes	Yes	NS	LB^&^	1 cm	Yes	No	No	NS	No
Machado et al., 2015 [15]	2015	1	1	0	1/0	Yes	No	Bipolar forceps	S4b-5	—	Yes	No	Yes	No	No
Yoon et al., 2015 [22]	2004–2014	45	32	1/32	0/32	Yes	Yes	NS	LB	2 mm^*ç*^	Yes	—	No	No	No
Shirobe and Maruyama, 2015 [23]	2001–2013	11	11	0	7/4	6/11^∧^	Yes	Harmonic scalpel, BiClamp	LB	1 cm	Yes	No	Yes	No	Yes (2)
Agarwal et al., 2015 [26]	2011–2013	24 (versus 46 open)	24	0	4/20	Yes	No	Harmonic scalpel, ultrasonic aspirator	S4b-5	—	Yes	4/4	No	No	No
Itano et al., 2015^#^ [27]	2007–2013	19 (versus 14 open)	16	0	0/16	Yes	Yes	Harmonic scalpel	LB	1 cm	Yes	—	No	No	No
Palanisamy et al., 2016 [24]	2008–2013	14	14	0	0/14	Yes	No	Harmonic scalpel, ligasure, ultrasonic aspirator	S4b-5	—	Yes	—	Yes	No	No

MiRC: minimally invasive radical cholecystectomy, RC: radical cholecystectomy, GbC: gallbladder cancer, LIOUS: laparoscopic intraoperative ultrasonography, LB: liver bed, NS: not specified, and S4b-5: liver segments 4b + 5.

^*∗*^Patients who underwent only laparoscopic exploration were excluded from the current analysis.

^*£*^Robot-assisted radical cholecystectomies.

^#^Multicenter retrospective study.

^&^Two patients underwent extended right hepatectomy.

^*ç*^Data reported in a previous paper from the same study group [28].

^∧^Liver bed resection not performed if tumor located on the peritoneal side of the gallbladder.

**Table 4:** MiRC for GbC: intraoperative and postoperative outcomes and pathologic findings.

Author, year of publication	Mean blood loss, mL (range)	Mean operation duration, min (range)	Morbidity	Mortality	Mean LOS, days (range)	Mean f-up duration, months	DFS at last f-up, rate	Recurrence location	T0-T1a/T1b/T2/T3	Mean number of resected nodes, (range)	NX/N0/N+
Cho et al., 2008 [16]	Minor	102 (89–146)	0	0	5 (4–7)	15	100%	—	1/0/2/0	4 (4-5)	0/3/0
de Aretxabala et al., 2010 [17]	NS	NS	0	0	3 NS	22	80%	Peritoneum (case 1)	3/13/5/2^$^	6 (3–12)	8/14/1^$^
Gumbs and Hoffman, 2010^§^ [18]	117 (50–200)	227 (120–360)	0	0	4 (3–5)	NS	NS	NS	0/1/0/0	3 (1–6)	0/1/0
Gumbs and Hoffman, 2010^§^ [19]	137 (50–300)	204 (95–360)	0	0	4 (3-4)	NS	NS	NS	0/0/0/1	3 (1–6)	0/0/1
Belli et al., 2011 [25]	85	162	0	0	5	33	100%	No	NA	NA	NA
Shen et al., 2012^*£*^ [20]	210 (50–400)	200 (120–360)	0	0	7	11	80%	NS	0/0/2/3	9 (3–11)	0/2/3
Gumbs et al., 2013^#^ [21]	160 (0–400)	220 (120–480)	0^&^	0	4 (2–8)	23	87%	Hep pedicle (case 1) Liver (case 2)	0/8/4/3	4 (1–11)	0/12/3
Machado et al., 2015 [15]	240	300	0	0	4	12	100%	—	0/1/0/0	9	0/1/0
Yoon et al., 2015 [22]	100 (10–1500)	205 (90–360)	6/32	0	4 (2–13)	60	95%	Liver (case 1) Lung (case 2) Liver + lung (case 3) Colon (case 4)	0/7/25/0	7 (1–15)	0/27/5
Shirobe and Maruyama, 2015 [23]	92 (10–643)	196 (150–490)	1/11	0	6 (4–19)	84	82%	Hep pedicle + pleural (case 1) Hep pedicle (case 2)	0/3/8/0	13 (9–18)	0/11/0
Agarwal et al., 2015^*∗*^ [26]	200 (100–850)	270 (180–340)	3/24	0	5 (3–16)	18	96%	Hep pedicle (case 1)	4/1/11/8	10 (4–31)	0/19/5
Itano et al., 2015^%^ [27]	152 (±90)	368 (±73)	1/16	0	9 (±1.6)	37	100%	—	1/2/13/0	13 (±3.1)	0/16/0
Palanisamy et al., 2016^%^ [24]	196 (±73)	212 (±26)	4/14	0	5 (±0.9)	51	75%	NS	0/0/11/1	8 (4–14)	0/9/3

MiRC: minimally invasive radical cholecystectomy, GbC: gallbladder cancer, f-up: follow-up, DFS: disease-free survival, NS: not specified, and LOS: length of hospital stay.

^§^Pathologic findings available for only one patient.

^#^Multicenter retrospective study.

^$^Data regarding 23 patients affected by GbC initially included in the study (including 5 patients who underwent only laparoscopic exploration, without attempt for MiRC).

^&^ The absence of postoperative bile leak, abdominal collection, and need for reoperation.

^*∗*^Blood loss, operation duration, LOS, and resected nodes # are expressed in median (range).

^%^Blood loss, operation duration, LOS, and resected nodes # are expressed in mean (±standard deviation). Only 12 patients affected by GbC were included in pathological and survival analysis.

## References

[1] Clinical Outcomes of Surgical Therapy Study Group (2004). A comparison of laparoscopically assisted and open colectomy for colon cancer. *The New England Journal of Medicine*.

[2] Colon Cancer Laparoscopic or Open Resection Study Group, Buunen M., Veldkamp R. (2009). Survival after laparoscopic surgery versus open surgery for colon cancer: long-term outcome of a randomised clinical trial. *Lancet Oncology*.

[3] Weiland S. T., Mahvi D. M., Niederhuber J. E. (2002). Should suspected early gallbladder cancer be treated laparoscopically?. *Journal of Gastrointestinal Surgery*.

[4] Jin K., Lan H., Zhu T., He K., Teng L. (2011). Gallbladder carcinoma incidentally encountered during laparoscopic cholecystectomy: how to deal with it. *Clinical and Translational Oncology*.

[5] Misra M. C., Guleria S. (2006). Management of cancer gallbladder found as a surprise on a resected gallbladder specimen. *Journal of Surgical Oncology*.

[6] Toyonaga T., Chijiiwa K., Nakano K. (2003). Completion radical surgery after cholecystectomy for accidentally undiagnosed gallbladder carcinoma. *World Journal of Surgery*.

[7] Jang J. Y., Heo J. S., Han Y. (2016). Impact of type of surgery on survival outcome in patients with early gallbladder cancer in the era of minimally invasive surgery. Oncologic Safety of Laparoscopic Surgery. *Medicine*.

[8] Dixon E., Vollmer C. M., Sahajpal A. (2005). An aggressive surgical approach leads to improved survival in patients with gallbladder cancer: a 12-year study at a North American Center. *Annals of Surgery*.

[9] Kondo S., Takada T., Miyazaki M. (2008). Guidelines for the management of biliary tract and ampullary carcinomas: surgical treatment. *Journal of Hepato-Biliary-Pancreatic Surgery*.

[10] Ito H., Ito K., D'Angelica M. (2011). Accurate staging for gallbladder cancer: implications for surgical therapy and pathological assessment. *Annals of Surgery*.

[11] Choi S. B., Han H. J., Kim C. Y. (2012). Fourteen year surgical experience of gallbladder cancer: validity of curative resect. *Hepatogastroenterology*.

[12] Aloia T. A., Járufe N., Javle M. (2015). Gallbladder Cancer: expert consensus statement. *HPB*.

[13] Mahid S. S., Hornung C. A., Minor K. S., Turina M., Galandiuk S. (2006). Systematic reviews and meta-analysis for the surgeon scientist. *British Journal of Surgery*.

[14] Moher D., Shamseer L., Clarke M. (2015). Preferred reporting items for systematic review and meta-analysis protocols (PRISMA-P) 2015 statement. *Systematic Reviews*.

[15] Machado M. A., Makdissi F. F., Surjan R. C. (2015). Totally Laparoscopic Hepatic Bisegmentectomy (s4b+s5) and Hilar Lymphadenectomy for Incidental Gallbladder Cancer. *Annals of Surgical Oncology*.

[16] Cho A., Yamamoto H., Nagata M. (2008). Total laparoscopic resection of the gallbladder together with the gallbladder bed. *Journal of Hepato-Biliary-Pancreatic Surgery*.

[17] de Aretxabala X., Leon J., Hepp J., Maluenda F., Roa I. (2010). Gallbladder cancer: role of laparoscopy in the management of potentially resectable tumors. *Surgical Endoscopy and Other Interventional Techniques*.

[18] Gumbs A. A., Hoffman J. P. (2010). Laparoscopic completion radical cholecystectomy for T2 gallbladder cancer. *Surgical Endoscopy*.

[19] Gumbs A. A., Hoffman J. P. (2010). Laparoscopic radical cholecystectomy and Roux-en-Y choledochojejunostomy for gallbladder cancer. *Surgical Endoscopy and Other Interventional Techniques*.

[20] Shen B. Y., Zhan Q., Deng X. X. (2012). Radical resection of gallbladder cancer: could it be robotic?. *Surgical Endoscopy*.

[21] Gumbs A. A., Jarufe N., Gayet B. (2013). Minimally invasive approaches to extrapancreatic cholangiocarcinoma. *Surgical Endoscopy*.

[22] Yoon Y.-S., Han H.-S., Cho J. Y. (2015). Is laparoscopy contraindicated for gallbladder cancer? A 10-year prospective cohort study. *Journal of the American College of Surgeons*.

[23] Shirobe T., Maruyama S. (2015). Laparoscopic radical cholecystectomy with lymph node dissection for gallbladder carcinoma. *Surgical Endoscopy*.

[24] Palanisamy S., Patel N., Sabnis S. (2016). Laparoscopic radical cholecystectomy for suspected early gall bladder carcinoma: thinking beyond convention. *Surgical Endoscopy*.

[25] Belli G., Cioffi L., D'Agostino A. (2011). Revision surgery for incidentally detected early gallbladder cancer in laparoscopic era. *Journal of Laparoendoscopic & Advanced Surgical Techniques*.

[26] Agarwal A. K., Javed A., Kalayarasan R., Sakhuja P. (2015). Minimally invasive versus the conventional open surgical approach of a radical cholecystectomy for gallbladder cancer: a retrospective comparative study. *HPB*.

[27] Itano O., Oshima G., Minagawa T. (2015). Novel strategy for laparoscopic treatment of pT2 gallbladder carcinoma. *Surgical Endoscopy and Other Interventional Techniques*.

[28] Cho J. Y., Han H.-S., Yoon Y.-S., Ahn K. S., Kim Y.-H., Lee K.-H. (2010). Laparoscopic approach for suspected early-stage gallbladder carcinoma. *Archives of Surgery*.

[29] Koshenkov V. P., Koru-Sengul T., Franceschi D., Dipasco P. J., Rodgers S. E. (2013). Predictors of incidental gallbladder cancer in patients undergoing cholecystectomy for benign gallbladder disease. *Journal of Surgical Oncology*.

[30] Chan K.-M., Yeh T.-S., Jan Y.-Y., Chen M.-F. (2006). Laparoscopic cholecystectomy for early gallbladder carcinoma: long-term outcome in comparison with conventional open cholecystectomy. *Surgical Endoscopy*.

[31] Sun C. D., Zhang B. Y., Wu L. Q., Lee W. J. (2005). Laparoscopic cholecystectomy for treatment of unexpected early-stage gallbladder cancer. *Journal of Surgical Oncology*.

[32] Cavallaro A., Piccolo G., Panebianco V. (2012). Incidental gallbladder cancer during laparoscopic cholecystectomy: managing an unexpected finding. *World Journal of Gastroenterology*.

[33] Schaeff B., Paolucci V., Thomopoulos J. (1998). Port site recurrences after laparoscopic surgery. A review. *Digestive Surgery*.

[34] Ohmura Y., Yokoyama N., Tanada M., Takiyama W., Takashima S. (1999). Port site recurrence of unexpected gallbladder carcinoma after a laparoscopic cholecystectomy: report of a case. *Surgery Today*.

[35] Z'graggen K., Birrer S., Maurer C. A., Wehrli H., Klaiber C., Baer H. U. (1998). Incidence of port site recurrence after laparoscopic cholecystectomy for preoperatively unsuspected gallbladder carcinoma. *Surgery*.

[36] Lee J.-M., Kim B.-W., Kim W. H., Wang H.-J., Kim M. W. (2011). Clinical implication of bile spillage in patients undergoing laparoscopic cholecystectomy for gallbladder cancer. *The American Surgeon*.

[37] Yamamoto H., Hayakawa N., Kitagawa Y. (2005). Unsuspected gallbladder carcinoma after laparoscopic cholecystectomy. *Journal of Hepato-Biliary-Pancreatic Surgery*.

[38] Shirai Y., Yoshida K., Tsukada K., Muto T. (1992). Inapparent carcinoma of the gallbladder. An appraisal of a radical second operation after simple cholecystectomy. *Annals of Surgery*.

[39] De Aretxabala X., Roa I., Burgos L. (2006). Gallbladder cancer: an analysis of a series of 139 patients with invasion restricted to the subserosal layer. *Journal of Gastrointestinal Surgery*.

[40] Evrard S., Falkenrodt A., Park A., Tassetti V., Mutter D., Marescaux J. (1997). Influence of CO_2_ pneumoperitoneum on systemic and peritoneal cell-mediated immunity. *World Journal of Surgery*.

[41] Varshney S., Buttirini G., Gupta R. (2002). Incidental carcinoma of the gallbladder. *European Journal of Surgical Oncology*.

[42] Paolucci V. (2001). Port site recurrences after laparoscopic cholecystectomy. *Journal of Hepato-Biliary-Pancreatic Surgery*.

[43] Drouard F., Delamarre J., Capron J.-P. (1991). Cutaneous seeding of gallbladder cancer after laparoscopic cholecystectomy. *The New England Journal of Medicine*.

[44] Neuhaus S. J., Texler M., Hewett P. J., Watson D. I. (1998). Port-site metastases following laparoscopic surgery. *British Journal of Surgery*.

[45] Itano O., Ikoma N., Takei H., Oshima G., Kitagawa Y. (1097). The superficial precoagulation, sealing, and transection method: a ‘bloodless’ and ‘ecofriendly’ laparoscopic liver transection technique. *Surgical Laparoscopic Endoscopic Percutaneous Technology*.

[46] Han H. S., Yoon Y. S., Cho J. Y., Hwang D. W. (2013). Laparoscopic liver resection for hepatocellular carcinoma: korean experiences. *Liver Cancer*.

[47] Nguyen K. T., Gamblin T. C., Geller D. A. (2009). World review of laparoscopic liver resection-2,804 patients. *Annals of Surgery*.

[48] Negi S. S., Singh A., Chaudhary A. (2011). Lymph nodal involvement as prognostic factor in gallbladder cancer: location, count or ratio?. *Journal of Gastrointestinal Surgery*.

